# Exploring Fetal Sex Dimorphism in the Risk Factors of Gestational Diabetes Mellitus—A Prospective Cohort Study

**DOI:** 10.3389/fendo.2019.00848

**Published:** 2019-12-06

**Authors:** Wen-Juan Wang, Lin Zhang, Dan-Li Zhang, Tao Zheng, Hua He, Fang Fang, Jun Zhang, Fengxiu Ouyang, Zhong-Cheng Luo, Shanghai Birth Cohort Study

**Affiliations:** ^1^Ministry of Education and Shanghai Key Laboratory, Department of Pediatrics, Xinhua Hospital, Shanghai Jiao Tong University School of Medicine, Shanghai, China; ^2^Department of Obstetrics and Gynecology, Prosserman Centre for Population Health Research, Lunenfeld-Tanenbaum Research Institute, Mount Sinai Hospital, Institute of Health Policy, Management and Evaluation, University of Toronto, Toronto, ON, Canada; ^3^Department of Obstetrics and Gynecology, Xinhua Hospital, Shanghai Jiao Tong University School of Medicine, Shanghai, China

**Keywords:** gestational diabetes mellitus, fetal sex, risk factor, prospective cohort, Chinese

## Abstract

Gestational diabetes mellitus (GDM) is a common pregnancy complication. Its etiology remains incompletely understood. Studies in recent years suggest that fetal sex may affect maternal metabolic milieu during pregnancy. We sought to assess whether there is fetal sex dimorphism in the risk factors of GDM. In a prospective pregnancy cohort in Shanghai, China, we studied 2,435 singleton pregnant women without pre-existing diabetes. GDM was diagnosed according to the International Association of Diabetes and Pregnancy Study Groups (IADPSG)' criteria. Log-binomial models were applied to obtain the adjusted relative risk (aRR). A total of 380 (15.6%) women developed GDM. Family history of diabetes was associated with an increased risk of GDM in women bearing a female fetus [aRR 1.74 (1.27–2.40), *p* < 0.001], but not in women bearing a male fetus (*p* = 0.68) (test for interaction, *p* = 0.03). Alcohol drinking was associated with an increased risk of GDM in women bearing a male fetus only (*p* = 0.023), although the test for interaction did not reach statistical significance (*p* = 0.055). In conclusion, family history of diabetes was associated with an increased risk of GDM in women bearing a female fetus only in this Chinese pregnancy cohort. There may be a need to consider fetal sex dimorphism in evaluating the risk factors of GDM.

## Introduction

Gestational diabetes mellitus (GDM) is a common pregnancy complication characterized by impaired glucose tolerance with first-time recognition during pregnancy (1). Major risk factors of GDM include pre-pregnancy overweight and obesity, excessive gestational weight gain (GWG) during early/mid pregnancy, certain ethnicity (e.g., Asian), advanced maternal age, multiple pregnancy, history of GDM and family history of diabetes (2–5). It has been recognized in recent years that fetal sex may influence glucose metabolism in the mother during pregnancy. Women bearing a female fetus may have a higher insulin resistance, but those bearing a male fetus may have a lower beta cell function during pregnancy (6, 7). A meta-analysis has shown that pregnant women carrying a boy have a 4% higher relative risk of GDM than those carrying a girl (8). However, among women with GDM, those who carried a girl have about 6% higher relative risk of developing type 2 diabetes in the future (9, 10). During pregnancy, the placenta secretes “diabetogenic” hormones (e.g., placental lactogen) into the maternal circulation, and higher maternal circulating concentrations of such diabetogenic hormones have been reported in women bearing a female fetus (11, 12). Taken together, available evidence suggests a possible fetal sex dimorphism in the maternal endocrine environment that may allow for differential effects of certain GDM risk factors. We sought to test this hypothesis in a prospective pregnancy cohort.

## Methods

This was a prospective cohort study based on the recently developed Shanghai Birth Cohort (SBC) (13). Briefly, the SBC cohort recruited women in pre-conception care (1/4) or early antenatal care (11–17 weeks of gestation; 3/4) clinics in 6 tertiary obstetric care hospitals in Shanghai between 2013 and 2015. The study was approved by the research ethics boards of Shanghai Xinhua Hospital (the coordination center, approved on August 23, 2013, ref no. M2013-010) and all participating hospitals. All study participants signed an informed consent form. As part of the SBC cohort, the present study sought to assess sex dimorphism in the risk factors of GDM in women bearing a singleton fetus with complete data on major risk factors of GDM (age, pre-pregnancy overweight/obesity status, weight gain in early pregnancy, history of GDM, and family history of diabetes) and without pre-gestational diabetes (normal fasting blood glucose levels during the first antenatal care visit). A total of 2,435 singleton pregnant women were eligible and included.

The primary outcome was GDM. All women received a 75 g oral glucose tolerance test (OGTT) in screening for GDM during 24–28 weeks of gestation. GDM was diagnosed according to the criteria proposed by the International Association of Diabetes and Pregnancy Study Groups (IADPSG): if any one of the glucose values was at or above the following thresholds: fasting 5.1 mmol/L, 1 h 10.0 mmol/L and 2 h 8.5 mmol/L (14).

Pre-pregnancy weight (kg) was based on self-reports, while weight at the early pregnancy visit (11–17 weeks) was measured to the nearest 0.1 kg using the routinely available electronic weighing device in the hospital's prenatal care clinics. Weight at the delivery visit was abstracted from the patient's medical chart. Height (cm) was measured to the nearest 0.1 cm using the routinely available electronic stadiometer in the prenatal care clinics. Pre-pregnancy BMI (kg/m^2^) was used to define weight status: underweight (<18.5), normal weight (18.5–23.9), overweight (24.0–27.9) and obese (≥28) according to Chinese standards.

GWG in early pregnancy was calculated as the difference between weight at the first antenatal visit (around 14 weeks; 11–17 weeks) and pre-pregnancy weight. GWG in early pregnancy and its velocity (kg/week) were standardized into Z scores, using BMI category-specific mean and SD values derived from the study cohort (because there were no standards for GWG in early pregnancy). The Z scores were obtained by subtracting the mean from an individual's raw value and then dividing by the SD. Because weight gain in late gestation in women with GDM would have been affected by clinical management after the diagnosis of GDM, the analysis on GWG as a risk factor was restricted to GWG in early pregnancy (before the clinical manifestation of the disease). Exploratory analyses observed similar findings for GWG z score and GWG velocity z score in early pregnancy; we presented the main results for GWG velocity in early pregnancy.

Ethnicity and family history of diabetes (first-degree relatives) were based on self-reports. Obstetric histories (parity, history of GDM) were based on self-reports and cross-verified in medical charts. Maternal age at recruitment was based on the date of birth and verified by personal identification card (with date of birth), and was classified as >35 years or not since maternal age >35 years is a classical risk factor of GDM (7). Smoking and alcohol drinking were based on self-reports in the early pregnancy (11–17 weeks) visit (did you smoking during this pregnancy? yes/no; did you drink alcohol during this pregnancy: yes/no).

Data were presented as mean±SD or number (%). Student *t*-test and Chi-square test were used to examine the differences in continuous and categorical variables, respectively. Log-binomial regression models were fitted to obtain the crude and adjusted relative risk (aRR) with 95% confidence intervals (CI) for the associations between risk factors and GDM. In the adjusted analyses, only co-variables with *p* < 0.2 were retained in the final parsimonious regression models to obtain more stable effect estimates. Interaction between two variables were assessed by examining the significance (*p*-value) of their multiplicative term in regression models. The study had a power of 93% to detect a significant interaction of fetal sex with a GDM risk factor of >=10% prevalence if there was a RR of >=2.0 in one stratum vs. the absence of relative risk increase in the other stratum, and a power of 47% if the RR was >=1.5, assuming a baseline event rate of 15% in GDM. All analyses were performed using Statistical Analysis System, Version 9.4 (SAS Institutes, Cary, North Carolina). *P* < 0.05 was considered statistically significant.

## Results

Of the 2,435 singleton pregnant women in the study cohort, 1,271 (52.2%) women had a male fetus, 1,164 (47.8%) had a female fetus, and 380 (15.6%) developed GDM. A greater proportion of women bearing a male fetus were at advanced maternity age (>35 years) (*p* = 0.002), or had gestational hypertension disorders (*p* = 0.013) than women bearing a female fetus (Table 1). All other risk factors (such as maternal overweight and obesity, history of GDM, family history of diabetes) were similar in women bearing a male vs. a female fetus. Average GWG and average GWG velocity in early pregnancy were all similar in women bearing a male fetus vs. those bearing a female fetus.

**Table 1:** Characteristics of a singleton pregnancy cohort (*n* = 2435) by fetal sex in Shanghai, China.

**Characteristic**	**Male (*n =* 1271)**	**Female (*n =* 1164)**	***P*^**a**^**
GDM	190 (14.9)	190 (16.3)	0.371
Mother age (year)	29.3 ± 3.8	29.0 ± 3.6	0.034
> 35 (yes)	117 (9.2)	69 (5.9)	0.003
Race, Han	1255 (98.7)	1149 (98.7)	1.000
Smoking	4 (0.3)	4 (0.3)	1.000
Drinking	120 (9.4)	111 (9.5)	0.945
Family history of diabetes	124 (9.8)	103 (8.9)	0.485
Primiparous	1080 (85.3)	971 (83.8)	0.311
Education, university	806 (63.5)	749 (64.5)	0.612
History of GDM	9 (0.7)	7 (0.6)	0.806
Chronic hypertension	8 (0.6)	2 (0.2)	0.112
Hypertensive disorders	71 (5.6)	50 (4.3)	0.161
Gestational hypertension	54 (4.2)	28 (2.4)	0.013
Preeclampsia/eclampsia	17 (1.3)	22 (1.9)	0.333
Height (cm)	162.1 ± 4.9	162.1 ± 4. 9	0.827
Pre-pregnancy weight (kg)	56.3 ± 8.7	56.3 ± 8.7	0.908
Pre-pregnancy BMI (kg/m^2^)	21.4 ± 3.0	21.4 ± 3.0	0.998
Pre-pregnancy BMI category			0.885
Underweight (n = 352)	181 (14.2)	171 (14.7)	
Normal weight (n = 1692)	882 (69.4)	810 (69.6)	
Overweight/obese (n = 391)	208 (16.4)	183 (15.7)	
Early pregnancy visit			
Gestational age (week)	14.2 ± 1.6	14.2 ± 1.7	0.668
Weight (kg)	58.4 ± 9.0	58.3 ± 8.9	0.831
GWG (kg)	2.23 ± 2.93	2.18 ± 2.83	0.650
GWG velocity (kg/week)	0.16 ± 0.21	0.15 ± 0.20	0.567
Gestational age (week) at delivery	38.9 ± 1.6	39.1 ± 1.5	0.014
Weight (kg)	71.3 ± 9.6	71.0 ± 9.3	0.472
GWG (kg)	15.0 ± 4.8	14.8 ± 5.0	0.221
GWG velocity (kg/week)	0.39 ± 0.12	0.38 ± 0.13	0.118

Data presented are Mean ± SD or n (%). GDM, gestational diabetes mellitus; BMI, body mass index; GWG, gestational weight gain.

a *P-values for comparisons of the two groups in T-tests for continuous variables and Chi-square tests for categorical variables*.

In the whole study cohort, faster GWG in early pregnancy was associated with an increased risk of GDM (aRR = 1.12 per SD increase in GWG velocity, 95% CI 1.03–1.23, *p* = 0.010) (Table 2). Advanced maternal age (>35 years) was associated with an increased risk of GDM in the whole study cohort without the adjustment for co-variables (RR = 1.41, *P* = 0.024), but the association became statistically non-significant after the adjustment (*P* = 0.098). Compared to normal weight women, overweight and obese women were at an increased risk of GDM [aRR = 1.76 (1.44–2.51), *p* < 0.001]. As expected, history of GDM [aRR = 3.49 (2.34–5.20), *p* < 0.001] and family history of diabetes [aRR = 1.44 (1.12–1.85), *p* = 0.005] were significant risk factors of GDM. The association of alcohol drinking with GDM did not reach statistical significance (aRR = 1.30, *p* = 0.072) in the whole study cohort. Fetal sex was not associated with GDM (female vs. male: aRR = 1.12, *p* = 0.209).

**Table 2:** Risk factors of gestational diabetes mellitus (GDM).

**Risk factor**	**Crude RR** **(95% CI)**	***P***	**Adjusted**^**a**^ **RR (95% CI)**	***P***
Early GWG (z score)	1.13 (1.04–1.23)	0.006	1.14 (1.04–1.24)	0.004
GWG velocity (z score)	1.12 (1.02–1.22)	0.013	1.12 (1.03–1.23)	0.010
Maternal age >35 y	1.41 (1.05–1.89)	0.024	1.27 (0.96–1.69)	0.098
Pre-pregnancy BMI				
Underweight	0.70 (0.50–0.98)	0.038	0.71 (0.51–0.99)	0.046
Overweight/obese	1.81 (1.47–2.22)	<0.001	1.76 (1.44–2.15)	<0.001
Family history of diabetes	1.56 (1.21–2.03)	<0.001	1.44 (1.12–1.85)	0.005
History of GDM	3.68 (2.37–5.73)	<0.001	3.49 (2.34–5.21)	<0.001
Alcohol drinking	1.23 (0.93–1.64)	0.150	1.31 (0.98–1.74)	0.065

Early GWG, Weight gain between pre-pregnancy and study visit at 14.2 ± 1.6 weeks of gestation; BMI, body mass index.

a *The adjusted models included pre-pregnancy BMI, GWG in early pregnancy (z score, or velocity z score in separate models), maternal age (>35 y: yes/no), family history of diabetes, GDM history and alcohol drinking; other variables were not significant at p > 0.2 and did not affect the comparisons, and were not included*.

Table 3 presents the risk factors of GDM by fetal sex, and Table 4 presents the estimates (95% confidence intervals) of interaction effects of fetal sex with GDM risk factors. Family history of diabetes was associated with an increased risk of GDM in women bearing a female fetus [aRR = 1.74 (1.27–2.40), *p* < 0.001], but not in women bearing a male fetus (*p* = 0.680); the test for interaction was statistically significant (*p* = 0.030). Faster GWG velocity in early pregnancy was associated with an increased risk of GDM in women bearing a female fetus [aRR = 1.17 (1.03–1.33); *p* = 0.017], but not in women bearing a male fetus (*p* = 0.178), although the test for interaction did not reach statistical significance (*p* = 0.262). Similarly, advanced maternal age (>35 years) was associated with an increased risk of GDM [aRR = 1.64 (1.08–2.48), *p* = 0.019] in women bearing a female fetus only, although the test for interaction did not reach statistical significance (*p* = 0.246). In contrast, alcohol drinking was associated with an increased risk of GDM [aRR = 1.51 (1.06–2.16), *p* = 0.023] in women bearing a male fetus only, and the test for interaction was close to statistical significance (*p* = 0.055). The associations of pre-pregnancy overweight/obesity with GDM were similar in women bearing a male vs. a female fetus (data not shown). There were no significant interactions between other risk factors of GDM (all *p* > 0.3).

**Table 3:** Stratified analyses of risk factors with fetal sex-divergent associations with gestational diabetes mellitus (GDM).

**Risk factor**	**Crude** **RR (95% CI)**	***P***	**Adjusted**^**a**^ **RR (95% CI)**	***P***
Fetal sex–male (*n =* 1271)				
GWG velocity (Z score)	1.09 (0.96–1.23)	0.201	1.09 (0.96–1.23)	0.178
Family history of diabetes	1.18 (0.78–1.78)	0.435	1.08 (0.74–1.59)	0.680
Mother age (>35, yes/no)	1.26 (0.83–1.90)	0.273	1.05 (0.71–1.56)	0.800
Alcohol drinking	1.55 (1.07–2.24)	0.020	1.51 (1.06–2.16)	0.023
Fetal sex–female (*n =* 1164)				
GWG velocity (Z score)	1.15 (1.02–1.31)	0.024	1.17 (1.03–1.33)	0.017
Family history of diabetes	2.01 (1.45–2.79)	<0.001	1.74 (1.27–2.40)	<0.001
Mother age (>35, yes/no)	1.66 (1.09–2.53)	0.018	1.64 (1.08–2.48)	0.019
Alcohol drinking	0.94 (0.60–1.49)	0.800	0.97 (0.62–1.51)	0.878

a *The factors in the multi-variable log-binomial models were GWG velocity Z score, pre-pregnancy BMI (category), maternal age (>35, yes/no), alcohol drinking, GDM history and family history of diabetes. Other factors were not included since they were not significant at P > 0.2 and did not affect the comparisons. P-values in tests for interaction were: 0.262 for fetal sex and GWG velocity, 0.030 for fetal sex and family history of diabetes, 0.246 for fetal sex and advanced maternal age (>35, yes/no), and 0.055 for fetal sex and alcohol drinking. The number of GDM was 190 of 1,271 women carrying a male fetus, and 190 of 1,164 women carrying a female fetus*.

**Table 4:** Tests for interaction effects of fetal sex with risk factors of gestational diabetes mellitus (GDM).

**Interaction**	Estimate (95% CI)^*^	***P***
Fetal sex ^*^ GWG velocity (z score)	0.080 (−0.099, 0.258)	0.262
Fetal sex ^*^ family history of diabetes	0.535 (0.021, 1.050)	0.030
Fetal sex ^*^ maternal age (>35, yes/no)	0.354 (−0.220, 0.928)	0.246
Fetal sex ^*^ alcohol drinking	−0.520 (−1.099, 0.059)	0.055

* The effect estimates are for the effect (in log scale) of the interaction item (for example, for fetal sex = female and family history of diabetes = yes) from log-binomial models adjusted for the main effects and other co-variables as in Table 3.

*GWG, gestational weight gain*.

## Discussion

Our study is the first to explore fetal sex dimorphism in the risk factors of GDM. In this Chinese pregnancy cohort, an increased risk of GDM was observed for family history of diabetes in women bearing a female fetus only. There is also some weak evidence of fetal sex dimorphism for alcohol drinking in relation to the risk of GDM; a significant association was observed for one fetal sex only, but the tests for interactions did not reach statistical significance. The findings call for attention to fetal sex dimorphism in evaluating the risk factors of GDM.

Family history of diabetes is a well-known risk factor of GDM. We observed an increased risk of GDM in women bearing a female fetus only. Circulating placental lactogen and oestriol levels have been reported to be higher in women bearing a female fetus than in those bearing a male fetus (11, 12). We speculate that higher circulating levels of such diabetogenic hormones may “unmask” the genetic susceptibility to glucose intolerance in these women with a family history of diabetes bearing a female fetus.

Excessive or higher GWG during early pregnancy and advanced maternal age have been associated with an increased risk of GDM (15, 16). A significant risk increase was observed in women bearing a female fetus only, but the test for interaction did not reach statistical significance (*p* = 0.25), probably due to the study's lack of power to detect moderate interactions. There is insufficient evidence in sex dimorphic association for maternal age or GWG.

There have been conflicting data concerning the association between alcohol drinking and GDM. Some studies reported that alcohol drinking might decrease the risk of GDM (17, 18), while others reported an increased risk (19). In contrast, alcohol drinking was a significant risk factor for GDM in women bearing a male fetus only in our cohort, although the test for interaction did not reach statistical significance (*p* = 0.055). More studies in other cohorts/populations are needed to clarify the association.

Recent studies have shown that fetal sex may affect glucose metabolism in the mother. We did not detect the reported about 4% higher relative risk of GDM in women carrying a boy in a meta-analysis (8). This is not a surprise; *ad hoc* power calculation indicated that our study's power was only 8% to detect such a small risk difference. Women bearing a male fetus might be slightly and significantly more likely to develop GDM than women bearing a female fetus (7), but women with GDM in the first pregnancy were more likely to develop type 2 diabetes before second pregnancy if they delivered a female infant (9, 10). The placenta produces placental lactogen and other diabetogenic hormones (20, 21), and circulating levels of these diabetogenic hormones have been reported to be higher in women bearing a female fetus (11, 12). Insulin resistance has been reported to be higher in women bearing a female fetus (6), yet beta-cell function appears to be lower in women bearing a male fetus (7), although there is a lack of confirmative studies. Taken together, available data suggest that the fetal sex may have a complex impact on the maternal endocrine milieu which may allow sex dimorphism in the effects of certain risk factors of GDM.

Our study has strengths and limitations. The study population was a large prospective cohort of relatively homogenous ethnicity (>98% Han Chinese) with low likelihood of confounding by ethnic difference in the susceptibility to GDM. Limitations are the crude measure of alcohol drinking (yes/no only), and limited power to detect risk factors of relatively small effects. Secondly, our study did not cover some possible GDM risk factors such as physical activity, dietary intake, the use of micronutrient or other supplements, passive smoking and sleeping habit. Lastly, we should caution that *p-*values in different strata in stratified analyses might be not comparable due to different sample sizes. Also, the generalizability of the findings to other ethnic groups/populations is unclear and cannot be assumed without confirmation studies.

In conclusion, family history of diabetes was associated with an increased risk of GDM in women bearing a female fetus only in this Chinese pregnancy cohort. Fetal sex dimorphism should be considered in evaluating the risk factors of GDM.

## Data Availability Statement

The datasets generated for this study are available on request to the corresponding author.

## Ethics Statement

This study complies with the guidelines of the Declaration of Helsinki. Written informed consent has been obtained from each study participant. The study was approved by the research ethics boards of Shanghai Xinhua Hospital (the coordination center, approved on August 23, 2013, ref no. M2013-010).

## Shanghai Birth Cohort Study Members

Xiaoming Shen, Hong Huang, Kun Sun, Jun Zhang, Weiye Wang, Weiping Xu, Fengxiu Ouyang, Lin Zhang, Hong Zhu, Fei Li, Fang Fang, Hua He, Yu Dong, Chen Chen Zhou, Yin Huang, Jinsong Zhang, Chonghuai Yan, Lisong Shen and Yixiao Bao, Xinhua Hospital and Chongming Branch, Shanghai Jiao Tong University School of Medicine, Shanghai, China; Ying Tian, School of Public Health, Shanghai Jiao Tong University School of Medicine, Shanghai, China; Weiwei Chen, Huijuan Zhang, Yu-Na Guo, Xiao-Yi Huang, Chuanliang Tong and Jian Xu, International Peace Maternity and Child Hospital, Shanghai Jiao Tong University School of Medicine, Shanghai, China; Yiwen Zhang, Fang Jiang and Xiaodan Yu, Shanghai Children's Medical Center, Shanghai Jiao Tong University School of Medicine, Shanghai, China; Guangjun Yu and Jinjin Chen, Shanghai Children's Hospital, Shanghai, China; Yu Zhang, Renji Hospital, Shanghai Jiao Tong University School of Medicine, Shanghai, China; Xiaotian Li, Haidong Cheng and Qinying Zhang, Obstetrics and Gynecology Hospital, Fudan University, China Tao Duan and Jing Hua, Shanghai First Maternity and Infant Care Hospital, Tong Ji University, Shanghai, China; Mingqing Xu, Bio-X Institute, Shanghai Jiao Tong University School of Medicine, Shanghai, China; Hua Peng, Maternal and Child Health Institute of Yangpu District, Shanghai, China.

## Author Contributions

LZ, JZ, FO, and Z-CL conceived the study. W-JW, D-LZ, TZ, HH, FF, LZ, JZ, FO, and Z-CL contributed to the acquisition of the research data. W-JW, HH, and D-LZ conducted the data analysis. W-JW conducted the literature search and drafted the manuscript. Z-CL was the guarantor taking full responsibility for the work as a whole, including the study design, access to data, and the decision to submit and publish the manuscript. All authors contributed to data interpretation, revised the article critically for important intellectual content, and approved the final version for publication.

### Conflict of Interest

The authors declare that the research was conducted in the absence of any commercial or financial relationships that could be construed as a potential conflict of interest.
